# The Pernicious Tendencies of Rampant Organization

**Published:** 1907-11

**Authors:** 


### The Pernicious Tendencies of Rampant Organization.

In a moment of impulse St. Paul questioned the veracity of all men. Had his observation extended to their judgment, there would have been little or no occasion for his later self-condemnation, for

—since Adam made the first great mistake—mankind has ever been led astray by the seductive voice of the promoter.

Especially is this true with reference to the medical fraternity, and the United States is just now witnessing the most ambitious and dangerous movement tending to the exploitation of a humanitarian profession that this or any other country has ever seen.

Not many years ago a few medical men—shrewd and calculating, with a proselytized practitioner as the dominant factor—laid the plans for a complete re-organization of the American Medical Association. With consummate skill of a certain kind, this reorganization was accomplished and, without realizing it, the medical men of the country have been brought to give to these organizers absolute power of dictation concerning all questions pertaining to the practice of medicine. No matter how rabid, radical, or nihilistic may be the views of these erstwhile leaders, they stand forth as accepted medical doctrine solely by virtue of their perfected system of organized control and promotion.

The result of this whole plan and process of organization has been to eliminate the individual member as a factor in the direction of the policies and business of the Association. Never was this so evident as at the recent June meeting of the A. M. A. at Atlantic City. Offices were bargained for and traded; combinations were effected, and numerous deals were completed.

*But of the 3700 members present, out of a total Association membership not exceeding 28,000, less than 100 members were aware of the deals afoot, and less than 20 engineered them.*

As was said by one prominent physician—a member of the Association for years, and known as one of the country's leading sanitarians—"This present method of organization is all wrong. We are all outsiders. I like to come to the meetings and, if not quite a factor, at least to know what is going on and who are the leading candidates for office. I want a chance to condemn or commend the policies that my annual fees help to support and propagate. In plain language, I want to be something more than a mere subscriber to the *Journal*." These setiments were not confined to one man. They were repeated again and again by members from coast to coast.

In opposition to this sort of sentiment the argument has been advanced that the business of the Association is expedited; that a greater volume can be transacted, and that the elimination of general discussion gives the members more opportunity for attending to scientific work!

Ingenious, 'tis true; but there are countless members who feel that it is quite as important to consider, debate and help to determine the great questions of medical economics, legislation, education, etc., as it is to listen to the reading and share in the perfunctory discussion of a number of dogmatic, stereotyped papers which, in any case, are bound to appear in the *Journal* in due course.

The plain fact is that the members of the American Medical Association have been cleverly "trade-unionized." Already the boycott has been brought into action—as some of the insurance companies, medical publishers and pharmaceutical manufacturers can testify—and the very tangible evils of misdirected paternalism are becoming all too evident. Medicine, alas, is being converted into a trade and, though an attempt is smugly made to hide this fact by platitudes and protestations, the spirit of crafty commercialism is certainly being fostered. Already the cachet of the Red Cross button—like the union members' card—is being pushed for all it is worth, and the fateful day approaches when the doctor who does not wear this "badge of business brotherhood" will not be allowed "on the job" with the one who does!

This means the death of aspiration and ambition, the stultifying of independent thought, the obliteration of individuality and the loss forever of that originality and personal initiative on which scientific progress always has depended, and so must ever continue to do.

Where and what is to be the end of the whole movement?

Unless a change in the present plan of centralization of control soon takes place, the unavoidable result will be a complete loss of professional prestige, with a consequent decrease in medical efficiency and an inevitable decline of medical incomes. The physician will practice his "trade" automatically—bound not only by fixed hours of service and fixed fees, but by fixed methods of treatment and practice!

The public, alive to the fallacious character and pernicious possibilities of such a system will have none of it. Intelligent laymen know too well that the human body is a constantly varying organism—that disease is subject to too many phases—to trust their cases to automatic, routine methods of treatment. Instead, they will seek the medical practitioner who has perfected himself not only in naming disease, but in the art of interpreting it—the medical man who possesses the genius of treatment, no matter what he employs or how. They want the man who can and does think for himself about their particular cases at the time they seek and most need his advice.

As this character can hardly fit the loyal adherent and follower of the present A. M. A. "Sanhedrim," his place and income can not fail to shrink in direct ratio to the increase of his independent colleague's success. Pressed, therefore, by the stress of material needs, many will be obliged to renounce their allegiance to the "Medical Union Idea," and as renunciation of a belief is often apt to lead to extremes in the opposite direction, an increase in charlatanry and all its evils may be confidently expected to follow.

How unfortunate is all this! Why will not the earnest, honestly-ambitious members of the A. M. A. see the rocks upon which their whole organization is being carried? How can they stand by and see a splendid craft wrecked by men who are conspicuous for little or nothing that truly tends to elevate the legitimate practice of medicine? It is a pitiable reflection upon the medical men of America who have always guarded their professional standing, and have correspondingly prized their integrity as physicians, that they now allow their futures and reputations to be thus dominated by men who, unlike Cæsar's wife, are not above suspicion—"machine" votes of confidence to the contrary notwithstanding, and as will be recognized with regret in the Babylonic days of disaster by which the future of the Association can not fail to be marked under its present regime.

It is high time that a definite movement was afoot to avert the dangers that threaten. One has only to talk with physicians the country over, and to read the non-subsidized representatives of the medical press of the United States, to learn the growing sentiment against "boss rule" in professional affairs. In the interests of progressive medicine and the worthy members of a splendid profession, it is to be hoped that the day may not be far removed when what has been described by others as "the Chicago Octopus"—and by ourselves as "the 'Machine' control of the A. M. A."—shall be laid out "on the sands of time," its power gone forever and awaiting only the waves of oblivion to sweep it out of the ken of future generations.—*Editorial in American Medical Journalist.*

